# Discipline in the New Military Hospitals

**Published:** 1915-05-29

**Authors:** 


### DISCIPLINE IN THE NEW MILITARY HOSPITALS.

### Problems Arising from an Initial Mistake.

### TWO SUGGESTED REFORMS.

In a civil hospital the power of maintaining discipline rests ultimately upon the ability to discharge a patient guilty of gross or repeated breaches of the rules. In the permanent military hospitals no difficulty arises. The soldier finds himself under the same system as that he is accustomed to in the regiment, whilst the staff are men with full knowledge of military organisation, and trained in its application to the special problems of hospital administration. In the new military hospitals things are very different. In most cases they are administered by men with very little or no knowledge of the Army regulations. The number of experienced orderlies is comparatively small, so that most of these hospitals are supplied with recently joined members of the Royal Army Medical Corps, who are not only inexperienced in ward work, but also know nothing of the ordinary routine of military hospital administration. All men who live under a rigid system of regulations are quick to detect ignorance or inexperience in those entrusted with enforcing it, and to take advantage of any relaxation in the rules arising from such causes.

### The New Chiefs and their Difficulties.

There are other difficulties confronting the administrative heads of the new military hospitals. In civil hospitals discipline is greatly facilitated by the fact that most of the patients are seriously ill and confined to bed, and are discharged as soon as they are convalescent or able to attend as out-patients. In the military hospitals the reverse is the case. The men have to be kept until they are fit at any rate for light duty with their units, or can be transferred to convalescent homes. The result is that often more than half the inmates of a ward are able to be up all day. Many of them are men in good health suffering from slight wounds, from wounds of the arm, from minor ailments, and such diseases as scabies. The provision of relaxation for such patients is no easy matter, especially in hospitals in large towns where the grounds are extremely limited in extent so that open-air games and exercises cannot be arranged. In wet and cold weather the small day-rooms which are fully sufficient for the demands of the ordinary population of the hospital are inadequate, and the ward has to be used as a recreation room. The usual regulations forbidding smoking and insisting upon quiet in the wards have to be relaxed, so that the whole atmosphere of the hospital undergoes a change.

### The Thoughtless Public.

The difficulties of administration are increased by actions on the part of the public springing from entirely praiseworthy motives. The natural desire to do something for those who have been wounded in the public service, or are in training for the national defence, sometimes expresses itself in highly undesirable ways. Alcoholic liquors are smuggled into the hospital, and the situation of the building with its boundaries abutting on public thoroughfares often renders this easy, and difficult of detection, especially when a large proportion of the inmates are able to be up and in the grounds. The enforcement of a harsh and rigid system of discipline is not desirable, and is not in accordance with the national habit. On the other hand, too great relaxation of discipline is bad both for the mental and physical welfare of our wounded and sick soldiers. It is necessary to find the right mean between the two.

### The Primary Error.

This desirable mean is to be found in the organisation of the ordinary military hospitals, and what is wanted is to bring our new military hospitals into line with them. The initial mistake was made when the War Office decided, when it took over civil institutions, to leave the administration in the hands of those who had hitherto carried it on. The complete difference in the character of the work was overlooked. When only a few wards in a hospital are taken over the difficulties do not arise, or only arise in a minor degree, because the soldier finds himself in a community obeying a recognised routine, and the force of the example of the majority—the herd instinct—makes itself felt, and he naturally does as the others. When the inmates of the hospital consist only of soldiers the tendency is in the opposite direction, and towards rebellion against civil authority, and the mere granting of a commission does not convert the civilian into a soldier with the soldier's habit of thought and experience.

### The Better Alternative.

It would have been better to have mobilised the reserve of retired Army medical men of senior rank, and to have placed them in administrative control, leaving the actual medical and surgical work to be carried out by the existing medical staff. The administration is so distinct from the actual medical work that in most cases no friction would have arisen. There are, too, many former non-commissioned officers and men of the R.A.M.C., too old for active service, who would be invaluable as orderlies and military police. If these men could be brought back to their old work it would not only render the administration of the new hospitals more efficient, but would release many of the younger men for work in the field. Again, the public desire to do something for the wounded when in hospital should be encouraged and make harmless by the adoption of the plan to be followed at King George Hospital. All members of the public wishing to contribute to the comforts of the wounded patients are invited to address their gifts to the matron, when the whole of them will be pooled and distributed by the sisters. This plan obviates the dangers we have mentioned, and gives the public the completest guarantee of good results.

